# Analysis of the complete organellar genomes of the rockweed *Fucus spiralis* (Fucaceae, Phaeophyceae) supports its infraspecific recognition as *Fucus vesiculosus* var. *spiralis*

**DOI:** 10.1080/23802359.2018.1463829

**Published:** 2018-04-23

**Authors:** Alejandra Alvarez, Juan Anaya, Bibiana Arellano, Austin Bartlebaugh, Michael C. Capurro, Adriana Carrillo, Isaiah R. Chacon, Lizbeth Cordova, Bethany Corral, Melina DaSilva, Giselle Del Valle, Alexis Diaz, Isaac Diaz, Carlos Donate, Isabella Fusco, Brian Garcia, Janette Garcia, Christian Godoy, Victor Gonzalez, Megan Hertzog, Nicholas Horton, Jeffery R. Hughey, Eli R. Kallison, Rafael Lopez, Jennifer Martinez, Rene Martinez, Kianna Mendez, Marie Pacheco, Maria Ramirez, David M. Ramirez, Jennifer M. Rios, Franca Rossi, Jorge Rua, Alyssa Sanchez, Daniela Sanchez, Maria Sanchez, Karla Santos, Rosaura Sierra, Daniel Soto, Alicia Steinhardt, Jesus Tavarez, Mark Tupas, Rolando T. Valdez, Christian Vargas, Rudy Vargas, Frances L. Wong, Adrian Zamora, Hartnell College Genomics Group

**Affiliations:** Division of Mathematics, Science, and Engineering, Hartnell College, Salinas, CA, USA

**Keywords:** California, *Fucus spiralis*, *Fucus vesiculosus*, mitogenome, plastid genome

## Abstract

*Fucus spiralis* L. is a broadly distributed monoecious intertidal seaweed. The specific status of *F. spiralis* however is debatable. Here, we contribute to the bioinformatics and systematics of *F. spiralis* by analysing the complete mitochondrial and plastid genomes of a specimen from California, U.S.A. The *F. spiralis* mitogenome is 36,396 base pairs (bp) in length and contains 67 genes, and the plastid genome is 125,066 bp in length and contains 171 genes. The *F. spiralis* genomes are 99.7% and 99.8% similar in nucleotide sequence to *F. vesiculosus*, and support the revised classification of *F. spiralis* to *Fucus vesiculosus* var. *spiralis*.

*Fucus spiralis* is a common intertidal rockweed that occurs in the Atlantic and northeastern Pacific Oceans, and the Mediterranean Sea (Guiry and Guiry 2018). Hybridization studies demonstrate that *F. spiralis* forms reproductively successful hybrids with the closely related species *F. vesiculosus*, which exhibit no significant decrease in hybrid fertility (Kniep 1925; Burrows and Lodge 1951; Billard et al. 2005). Molecular phylogenetic analyses of the two species yield polytomies, and DNA sequences of accepted species markers find that *F. spiralis* differs from *F. vesiculosus* by as little as 0 bp for both *cox*1 and the internal transcribed spacer regions (Serrão et al. 1999; Coyer et al. 2006, 2011; Kucera and Saunders 2008; Laughinghouse et al. 2015). The only diagnostic feature appears to be highly polymorphic microsatellite markers (Engel et al. 2003; Wallace et al. 2004; Billard et al. 2005). In this study, we characterize the organellar genomes of *F. spiralis* to further understand its relationship to *F. vesiculosus*.

*Fucus spiralis* (Voucher Specimen – UC2050586) was collected from Pacific Grove, California (36°38′02.0″N, 121°56′19.7″W); its DNA was isolated following Lindstrom et al. (2011). The 150 bp paired-end library construction and sequencing was performed by myGenomics, LLC (Alpharetta, GA). The genomes were assembled by mapping the reads against *F. vesiculosus* (GenBank – FM957154, AY494079) with the Low Sensitivity/Fast setting in Geneious R11 (Biomatters Limited, Auckland, New Zealand) and annotated using Sequin software. The mitogenome was aligned with other Phaeophyceae using MAFFT (Katoh and Standley 2013). The RaxML analysis was executed using complete mitogenome sequences at Trex-online (Boc and Makarenkov 2012) with the GTR + gamma model and 1000 fast bootstraps, then visualized with TreeDyn 198.3 at Phylogeny.fr (Dereeper et al. 2008).

The *F. spiralis* mitogenome (GenBank – MG922856) is 36,396 bp in length and contains 3 rRNA, 26 tRNA, and 28 other protein-coding genes. Its gene content and organization are the same as *F. vesiculosus* (Oudot-Le Secq et al. 2006). *Fucus spiralis* differs in sequence from *F. vesiculosus* by only 114 nucleotide SNPs and 10 gaps (99.7% similar). Comparison of all 9,367 protein coding amino acids finds 18 amino acid substitutions between the two species, of which only nine are radical (=the physiochemical properties are altered). Phylogenetic analysis of *F. spiralis* positions it in a fully supported clade with *F. vesiculosus* (Figure 1). The plastid genome (GenBank – MG922855) is 125,066 bp in length and contains duplicate copies of 16S, 23S and 5S rRNAs, 26 tRNAs, and 139 protein-coding genes. It is also highly similar to *F. vesiculosus* in chromosomal content and structure (Le Corguillé et al. 2009). *Fucus spiralis* differs in sequence from *F. vesiculosus* by 234 nucleotide SNPs and 67 gaps (99.8% similar), and shows 168 amino acid substitutions out of 31,893 total amino acids, of which only 32 were radical.

**Figure 1.**
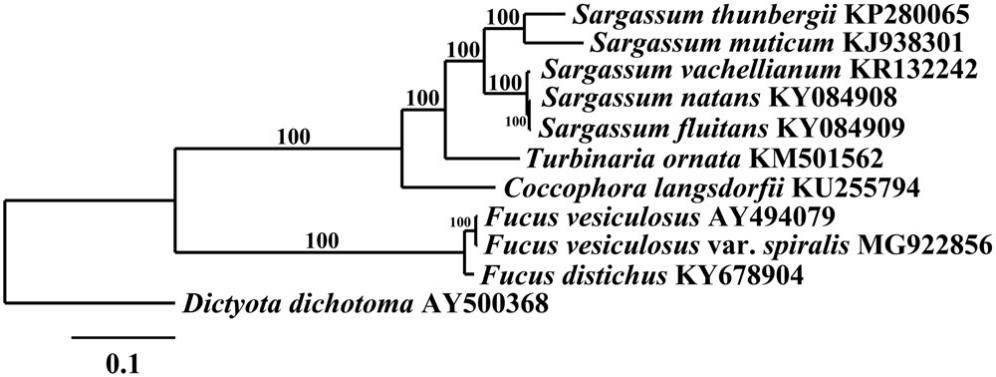
Maximum-likelihood phylogram of *Fucus vesiculosus* var. *spiralis* (MG922856) and related Phaeophyceae mitogenomes. Numbers along branches are RaxML bootstrap supports based on 1000 nreps. The legend below represents the scale for nucleotide substitutions.

On the basis of this genomic data and evidence from the biological, marker, and phylogenetic species concepts, we conclude that the name *F. spiralis* should be reduced to varietal status under *F. vesiculosus*, *F. vesiculosus* var. *spiralis* (Linnaeus) Roth.
